# Behaviour of one-step spray-coated carbon nanotube supercapacitor in ambient light harvester circuit with printed organic solar cell and electrochromic display

**DOI:** 10.1038/srep22967

**Published:** 2016-03-09

**Authors:** Sampo Tuukkanen, Marja Välimäki, Suvi Lehtimäki, Tiina Vuorinen, Donald Lupo

**Affiliations:** 1 Tampere University of Technology, Department of Automation Science and Engineering, Tampere, FI-33101, Finland; 2 VTT Technical Research Centre of Finland, P.O.Box 1100, FI-90570 Oulu, Finland; 3 Tampere University of Technology, Department of Electronics and Communications Engineering, Tampere, FI-33101, Finland

## Abstract

A printed energy harvesting and storage circuit powered by ambient office lighting and its use to power a printed display is reported. The autonomous device is composed of three printed electronic components: an organic photovoltaic module, a carbon-nanotubes-only supercapacitor and an electrochromic display element. Components are fabricated from safe and environmentally friendly materials, and have been fabricated using solution processing methods, which translate into low-cost and high-throughput manufacturing. A supercapacitor made of spray-coated carbon nanotube based ink and aqueous NaCl electrolyte was charged using a printed organic photovoltaic module exposed to office lighting conditions. The supercapacitor charging rate, self-discharge rate and display operation were studied in detail. The supercapacitor self-discharge rate was found to depend on the charging rate. The fully charged supercapacitor was used as a power source to run the electrochromic display over 50 times.

It is widely recognized that our present usage of non-renewable energy and raw materials is unsustainable. Continuing the current level of consumption of non-renewable resources will lead to pollution and other environmental damage, in addition to contributing to global climate change. It is critical for the future of the earth and society to develop low carbon emission energy sources as well as increase the use of environmentally friendly materials and processes for production of energy handling and storage devices. This is necessary not only for large-scale energy generation, but also for small-scale objects; fitting all of the predicted hundreds of billions of distributed smart objects for the Internet-of-Things with batteries is environmentally not a sustainable option.

Printed electronics and energy harvesting^1^ are potential ways to address the constantly growing need for advanced technologies that are also sustainable in energy and material consumption. Harvesting of energy from ambient sources enables autonomously powered ubiquitous intelligence for the Internet of Things and Cyber-Physical Systems. In addition, printed electronics provides a high-throughput method for manufacturing low-cost circuits which can be embedded everywhere in the built environment. Implementation of these advanced functionalities in the field of healthcare and well-being can improve the quality of life, while applications in process control and access monitoring provide services for industry.

Carbon nanomaterials, such as graphene^2^,^3^ and carbon nanotubes (CNT)^4^, have multiple desirable properties such as high electrical and thermal conductivity, high tensile strength, high surface area, chemical sensitivity, flexibility, transparency and low weight. Furthermore, these materials can be deposited using printing and coating techniques. The authors have previously demonstrated the feasibility of solution processed CNT and graphene films in various applications, such as stretchable electrodes on rubber^5^, piezoelectric sensors^6^,^7^ transparent touch panels^8^,^9^, and optoelectronics^10^.

Supercapacitors (SC), also known as ultracapacitors and electric double-layer capacitors (EDLC), are electrochemical energy storage devices which have higher cycle life and higher power output than batteries^11^,^12^,^13^. SCs are promising interim energy storage components for ubiquitous energy harvesting systems; they can be fabricated by printing methods from environmentally friendly materials and can be easily disposed of at the end of their life-cycle. Recently, there has been intensive research targeting conducting high surface area electrode materials for SCs, which could replace conventionally used and poorly conducting activated carbon materials. It has been shown that CNTs^14^,^15^,^16^ and graphene^17^,^16^ are promising candidates in the race for conductive high surface area materials.

In the recent literature, there are also a few demonstrations of solution processed CNT-only-SCs^18^,^19^,^20^,^21^,^22^. Previously, authors have studied SC fabrication using CNT based nanocomposite ink^25^,^26^,^27^ and graphene/conducting polymer composite^28^ as active electrode materials as well as the use of nanocellulose as separator^27^,^29^.

Light energy harvesting in general has been extensively studied in the literature^1^. There have also been interesting demonstrations of using light harvesting in combination with other harvesting sources in multi-source energy harvesting systems^31^,^32^,^33^. Although energy harvesting has been widely studied, the developed circuits are typically not fabricated from printed, flexible and low-cost components, which limits their suitability for low-cost mass production. The authors have recently demonstrated printed energy harvesting circuits gathering energy from an electromagnetic field using a printed radio frequency (RF) antenna^23^ and from movement using a piezoelectric sensor^24^, and used solution processed SCs as the energy storage devices.

In this paper, we evaluate the behaviour of a printable CNT based SC in an ambient light harvesting circuit. The SC was fabricated using one-step spray-coated CNT electrodes, which simultaneously function as active high surface area electrode and current collector. In earlier papers^24^,^25^,^26^, the authors demonstrated printable CNT based SCs using blade-coating technique in comparison to the spray coating used here. Very recently, the authors have used similar OPV and ECD components to demonstrate functionality of SC made of electrodeposited graphene/conducting polymer composite, printed graphine ink and metallic current collector^28^, but not studied the SC behaviour in detail. In this paper, we focus on SC performance in an energy-autonomous circuit with a printed organic photovoltaic (OPV) module and a printed electrochromic display (ECD) element. In particular, the charging of the SC with the OPV module under typical indoor lighting conditions, the SC self-discharging and the SC voltage evolution during the ECD operation have been investigated.

## Results and Discussion

### Carbon nanotube supercapacitor fabrication and characterization

**Figure 1:**
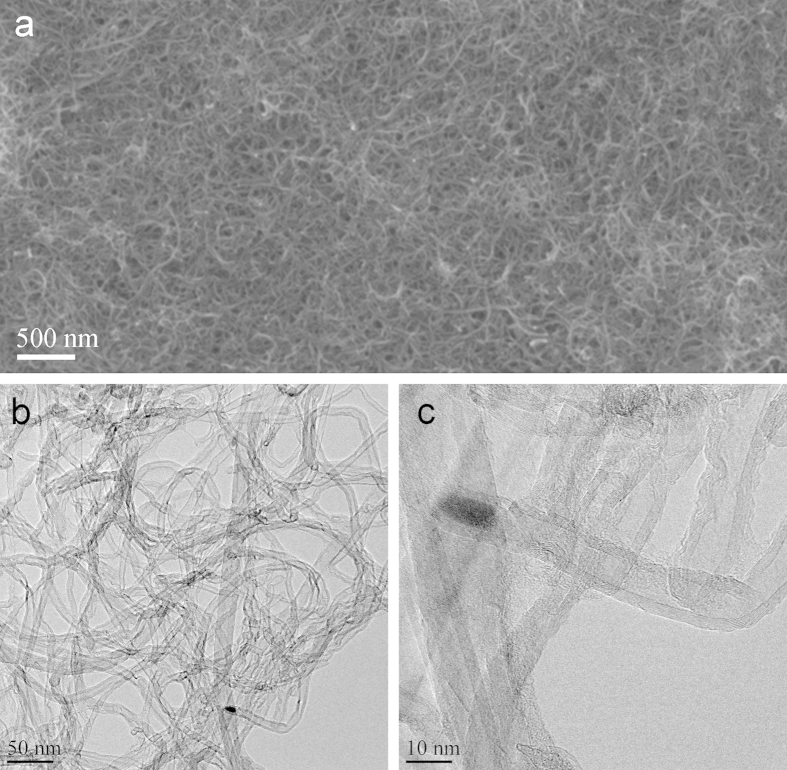
(**a**) SEM and (**b,c**) TEM images of CNT/xylan nanocomposite ink used in the supercapacitor electrode fabrication.

CNT electrodes for SC were prepared from a CNT/xylan nanocomposite ink, which works simultaneously as a high surface area active electrode material and electrically conducting current collector material. The composition of the CNT/xylan nanocomposite film can be seen from the scanning electron microscope (SEM) image in Fig. 1(a). The SEM image shows that CNTs are homogeneously distributed in the film. The transmission electron microscope (TEM) images in Fig. 1(b,c) show that the CNT ink contains multi-walled CNTs of diameter of about 10 nm with a very narrow diameter distribution. The images indicate that the CNTs are mostly bare; only a very thin dispersing agent (xylan) coverage can be observed in some of the tubes in the high magnification image Fig. 1(c). The CNT electrodes for SC were patterned on a plastic polyethylene terephthalate (PET) film by a spray-coating technique. The thickness of resulting electrodes was 6 ± 1 *μ*m. The sheet resistance of the CNT electrodes determined by a standard four-probe measurement was about 20 Ω/◻.

**Figure 2:**
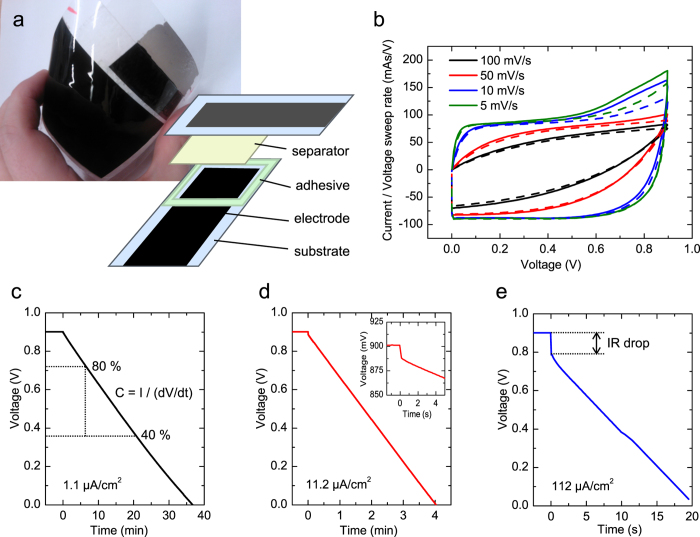
(**a**) A photograph of the fabricated supercapacitor and a schematic of its structure. (**b**) Cyclic voltammetry curves measured after the supercapacitor assembly (solid lines) and four months later (dashed lines). The current is divided by the sweep rate for each curve, giving essentially the capacitance. (**c,d**) Galvanostatic discharge curves with different discharge currents. The inset in (**d**) is an enlargement of the beginning of the discharge to show the IR drop.

The SC was assembled into a symmetric configuration by laminating two CNT electrodes on both sides of a paper separator soaked with NaCl aqueous electrolyte. The assembly was sealed using a two sided adhesive layer between the PET films. A photograph of the assembled SC is presented in Fig. 2(a).

The SC was electrically characterized using a Zennium electrochemical workstation (Zahner Elektrik GmbH). The cyclic voltammogram (CV) curves presented in Fig. 2(b) show that the SC has good qualitative capacitive behaviour. The quantitative electrical performance of the SC was obtained using galvanostatic discharge measurement according to an industrial standard IEC 62391-1^30^. The galvanostatic discharge curves are shown in Fig. 2(c,d). The capacitance is calculated from the slope of the curve between 80% and 40% of the initial potential; the ESR from the initial IR drop at the beginning of the discharge.

The results obtained with different discharge currents are summarized in Table 1. The SC capacitance (C) and equivalent series resistance (ESR) varied from 81 to 93 mF and from 37 to 46 Ω, respectively, depending on the application class. Accordingly, the area specific capacitance ranged from 2.7 to 3.1 mF/cm^2^ and the mass specific capacitance ranged from 1.5 to 1.7 F/g. The application class for the device under test defines the discharge measurement current as described in the measurement standard.

A leakage current of 14 A was measured after holding the SC for 30 min at a constant voltage bias of 0.9 V. Four such measurements were made with the SC completely discharged in between; the leakage current was initially 26 A but decreased to 14 A. This is likely due to the depletion of impurities which cause leakage through irreversible reactions.

The CV measurements were performed again after 5 months storage in ambient room conditions. The repeated measurements showed almost identical behaviour of the SC, as can be seen from Fig. 2(b). This implies that the fabricated aqueous CNT SC has a good storage life stability in ambient conditions. However, the SC electrodes were kept short-circuited during the storage, whereas aging tests are usually run under voltage and temperature stress. In comparison, activated carbon SCs underwent about 10% decrease in capacitance during 3-month aging tests^34^.

The capacitance obtained here corresponds to our previous work, where blade-coating deposition technique was used with a similar CNT/xylan ink^25^. However, spray-coating generally results in thinner films than blade-coating. The total surface area of the porous electrodes is expected to scale with the electrode thickness. In the previous work with CNT-based electrodes, the electrode thickness as well as the obtained area specific capacitance was twice of that obtained in this work (6 versus 3 mF/cm^2^).

In a very recent work, the area specific capacitance of 18 mF/cm^2^ and ESR of 20 Ω were obtained for PEDOT/rGO (i.e. a composite of poly(3,4-ethylenedioxythiophene and reduced graphene oxide) active electrode supercapacitors^28^. The use of metallic current collector explains a lower ESR than obtained in this work. The capacitance of the PEDOT/rGO supercapacitors was higher than CNT due to additional pseudocapacitance of the conducting polymer as well as a thicker active layer.

### Ambient light harvesting using organic photovoltaics

**Figure 3:**
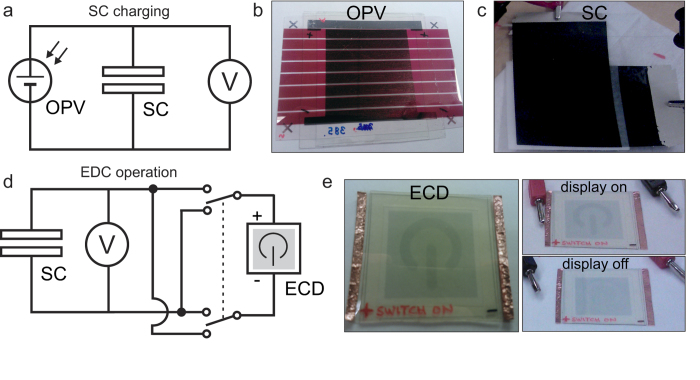
(**a**) The circuit used for the supercapacitor (SC) charging with the organic photovoltaic (OPV) module. Photographs of the (**b**) OPV module and (**c**) SC device. (**d**) The circuit used for the electrochromic display (ECD) operation powered by the charged SC. (**e**) Photographs of the ECD element and the comparison of its ON and OFF states (see Supplementary information Fig. S2).

The light energy was harvested from office room lighting using a printed organic photovoltaic (OPV) module which was previously reported^35^. The current-voltage (I-V) curves for two OPV modules used in this work are presented in Supplementary Information Fig. S1. The circuit for the light harvesting circuit is presented in Fig. 3(a) and the connected components in Fig. 3(b,c). The SC was charged under office lighting with a simultaneous monitoring of the SC voltage using a computer-controlled multimeter.

The ambient illuminance level was measured using a lux meter to define the luminous power prior to each harvesting experiment. The SC was charged by the OPV module 315 under three different lighting conditions; one by selecting an appropriate location in the office where the light comes from office ceiling lighting and the other two by placing the OPV module on two different distances (20 and 40 cm) from a fluorescent office desk lamp. The fabricated OPV module absorbs light between wavelengths 350 and 600 nm^36^ and the fluorescent lamp illuminates between 350 and 700 nm^37^, thus the condition for sufficient light absorption took place in the harvesting circuit studied here. The open circuit voltages of the OPV module under each of the lighting conditions are reported in Table 2.

**Figure 4:**
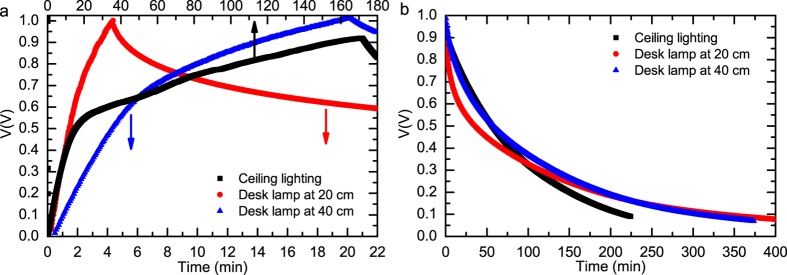
The SC voltage evolution (**a**) during the charging in the light harvesting experiment described in Fig. 3(a) in three different office lighting conditions and (**b**) the SC self-discharging curves when disconnected from the OPV module.

When the OPV module was connected to the uncharged SC, the output voltage dropped to zero and rose slowly while the SC was charged. The selected lighting conditions and charging times are presented in Table 2 and the charging curves are shown in Fig. 4(a). The targeted maximum voltage of the SC was about 1 V, which corresponds to the output of the OPV module into the SC load, but in the case of lowest illuminance levels the SC could only be charged to 0.92 V.

### Supercapacitor self-discharge behaviour

The EDLC has a tendency for relatively fast self-discharging, which is a themodynamically favorable process. Three typical self-discharging mechanisms are overcharging, side reactions and ohmic leakage^38^. The self-discharging of the SC was studied here in three cases where it was charged by the OPV module under three different lighting conditions (See Table 2).

After charging the SC up to the voltage 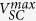 as shown in Fig. 4(a), it was disconnected from the OPV module 315 and allowed to self-discharge while the SC output was monitored with a high-impedance multimeter (input resistance >10 GΩ). These self-discharge times to 0.5 V and 0.1 V are shown in Table 2 and the SC self-discharge curves are plotted in Fig. 4(b).

The SC self-discharge timescales and shapes in Fig. 4(b) are relatively independent of the light intensity to which the OPV module was exposed. This is an advantage for this type of energy harvesting application, because the circuit can be easily designed for multiple light conditions. However, the SC self-discharge is somewhat faster in the case of higher illuminance under the desk lamp (at 20 cm vs. 40 cm distance). This can be explained by the limited speed of SC charging due to the timescale of the electric double layer arrangement^39^. This effect is even better visible in the following chapter when a fast current peak is drawn from the SC for ECD operation.

### Electrochromic display operation

The autonomously powered energy harvesting circuit used for ECD operation is presented in Fig. 3. The autonomous circuit operation was demonstrated by charging the SC with the OPV module 385 under a typical office lighting and subsequently using the SC to operate the ECD element.

The ambient light conditions were selected according to the European Standard for light and lighting of indoor work places (EN 12464-1:2002)^40^. Based on the standard, minimum illuminance of 300 lx is required for filing and copying, 500 lx for writing, typing, reading and data processing, and 750 lx for technical writing. Three different lighting conditions which are listed in Table 3 were used to study the autonomous circuit demonstrator operation.

**Figure 5:**
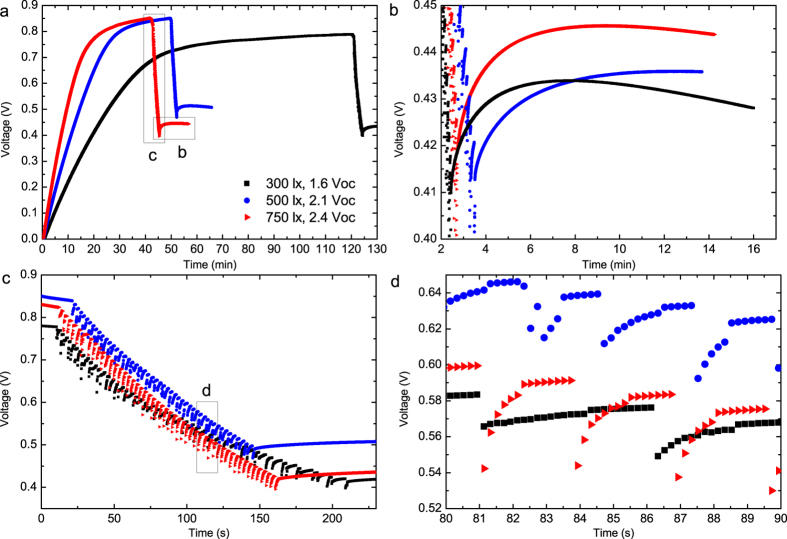
The evolution of SC voltage when charged with the OPV module under ambient lighting, and subsequent use of the SC for powering the ECD operation. (**a**) The overall SC voltage curves during charging and ECD operation. (**b**) Zoom-in to the voltage recovery related to re-organization in the electric double-layer of the SC after the ECD operation cycling was finished. (**c**) The SC voltage during the 100 cycles of ECD operations (50 times ON and 50 times OFF). (**d**) Zoom-in to the ECD ON and OFF cycling.

For the SC charging under different illumination levels, the OPV module was located at fixed distances below the fluorescent office lamp. The SC voltage profiles during the charging under three different lighting conditions and the subsequent EDC operations are presented in Fig. 5. After the SC was sufficiently charged, the OPV module was disconnected from the supercapacitor. The ECD display was then operated by changing its status between ON and OFF for 50 full cycles. Two different states of the ECD are shown in Fig. 3(e). The obtained ECD states correspond well to the ON and OFF states when operated using a standard voltage supply (shown in Supplementary information Fig. S2). After 50 cycles of ON-OFF display operations, the SC voltage had dropped to approximately 0.4–0.5 V, a voltage at which the ECD display contrast began to decrease.

One can see from the SC voltage curves in Fig. 5(d) that a “fast voltage recovery” takes place after a sudden voltage drop after each display update. A similar but even stronger effect is seen after the end of sequential display operation in Fig. 5(b). This effect is due to different time-scale effects in the SC, caused by the kinetics of the electric double-layer arrangement in the SC^39^. Fast current peaks are provided through ions in shallow pores, causing a sudden drop in the measured device voltage. However, the voltage is then recovered through ions from deeper in the pores.

## Conclusions

A supercapacitor (SC) from CNT-only electrodes was fabricated using methods compatible with low-cost and high-throughput mass manufacturing. The one-step spray-coated large-area (30 cm^2^) CNT-based SC fabricated in this work had a capacitance of 88 mF, resulting area specific capacitance of 3.0 mF/cm^2^ and mass specific capacitance of 1.6 F/g. This value is similar to values obtained in the recent literature, where CNT electrodes are used simultaneously as active electrodes and current collectors. The equivalent series resistance (ESR) was about 41 Ω, which is relatively low for a SC with CNT-only electrodes without a metallic current collectors.

The SC performance in an energy-autonomous circuit with a printed organic photovoltaic (OPV) module and a printed electrochromic display (ECD) was investigated, especially the charging of the SC under typical different ambient indoor lighting conditions, the SC self-discharge rate and the SC voltage evolution during the ECD operation. The assembled energy harvester circuit was suitable for effective charging of the SC with OPV module under office lighting. The study of SC self-discharge revealed that the SC undergoes a faster self-discharge process after a quick charging under high light intensity, in comparison to a slow charging with low ambient intensity lighting. This is explained by the filling of deep pores of the SC electrodes, which are not accessed in the fast charging process, where only easily available pores are filled with ions. The fully charged SC was able to operate the EDC over 50 times with a single charging cycle.

The CNT-based SC is a promising energy storage device for an autonomous light harvester circuit running the EDC. This type of energy harvesting has potential applications in Internet-of-Things and ubiquitous intelligence applications. Multi-source energy harvesting systems are of particular interest because it could provide power even when one of the harvesting sources is not available, for instance in solar harvesters during the night. For example, the combination of a light harvester circuit presented here with our recently developed piezoelectric kinetic energy harvester would have wide range of applications in the field of autonomously powered portable personal devices.

## Methods

### Supercapacitor fabrication and characterization

Supercapacitor electrodes were fabricated on a 125-m-thick poly(ethylene terephthalate) (PET) film (Melinex ST506 from DuPont Teijin Films). The PET substrate was cut into 12.5 cm × 9.5 cm pieces and the cut substrates were wiped with 2-propanol (IPA) prior to electrode fabrication.

Mechanical masks were used to define the rectangular (8.5 × 5.5 cm^2^) ink patterns. The rectangular shape masks were made from the PET film by carving them with a craft knife. To prevent the ink from leaking underneath the mask during spray coating, a temporary bonding adhesive (Zig 2-Way Glue) was used around the edges of the pattern to secure the PET mask in place.

A CNT based nanocomposite ink was used as electrode material in the supercapacitor. The same material layer worked simultaneously as an active layer and as a current collector. The CNT/xylan nanocomposite ink formulation was prepared by a collaborator (Morphona Ltd., Finland) similar way as in a previous study^25^. The CNTs were suspended in aqueous solution using xylan in its acidic form as a dispersion agent. The CNTs had an average diameter of 9.5 nm, an average length 1.5 *μ*m and a surface area of 250–300 m^2^/g (NC7000, purchased from Nanocyl). The ink contained 3 w-% multi-walled CNTs and 1.5 w-% xylan polymer. Total dry material content was 4.5 w-%. The mixing of CNTs and xylan was done by ultrasonication, using typically 10 min sonication with 100 W power for a 100 ml was 10 min with 100 W power. The SEM and TEM images of the ink are shown in Fig. 1.

Spray coating of the CNT/xylan nanocomposite ink was done manually with an airbrush (Silverline) which used compressed air (1 bar) as a carrier gas. Spray-coating was done on top of a 60 °C hot plate so that the hot plate would dry the ink just enough to ensure even layer formation. Without the hot plate, the ink aerosol would form large individual drops on the surface of the PET substrate. The hot plate dried the electrodes and no additional heat treatment was used. Masses for the two fabricated CNT electrodes were 41.6 and 44.5 mg.

The CNT/xylan nanocomposite electrode thickness was measured using a micrometer screw (Mitutoyo Absolute). The thicknesses was measured from five different points from both electrodes.The thickness of PET substrates were measured similarly and substracted from the elecrode value to get the final thickness.

The sheet resistance measurements for fabricated electrodes were performed using an in-house made four-probe setup and a Keithley multimeter compatible with the four-wire measurement. The four probes were set in a row with a 3 mm separation between adjacent probes. The DC current was supplied to the outer two probes while the voltage was measured from the inner ones (described in more detail elsewhere^8^).

The supercapacitor was assembled by sandwiching the electrodes with a separator paper (NKK TF4050) which was soaked in the electrolyte, 1 M NaCl (aq). The electrodes were positioned at a 90 angle to achieve an electrode overlap of 5.5 cm × 5.5 cm. The device was sealed with an adhesive film (UPM Raflatac) around the edges. Silver flake ink was applied to the electrode ends to ensure good contacts to the measurement device. The active area of the assemble device was 30.2 cm^2^. The total mass of CNT electrodes mass on only the active area (on both electrodes) was m = 55.7 mg, calculated assuming even layer thickness.

The measurements were conducted based on the specifications of standard IEC 62391-1 30. Initially, CV curves were recorded from 0 V to 0.9 V at rates 100, 50, 10 and 5 mV/s to obtain an initial view of device properties (1st cycle). The capacitance and ESR were determined from galvanostatic discharge experiments: the supercapacitor was charged to 0.9 V, held at that potential for 30 minutes, and then discharged with a constant current. The capacitance was calculated from the slope of the voltage decrease over time between 80% and 40% of the maximum voltage according to:


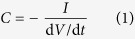


The ESR was determined from the initial IR drop of the voltage at the beginning of the discharge:


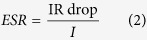


The discharge current depends on the capacitance and the application class; the current for ESR determination is 10 times that of the capacitance determination for a particular class. An initial mesurement was performed with discharge current 220 A (7.2 A/cm^2^) to obtain an initial value for the capacitance. Measurements were then carried out with 34 A (1.1 A/cm^2^) for Class 2, 340 A (11.2 A/cm^2^) for Class 3 and 3.4 mA (112.4 A/cm^2^) for Class 4. The ESR for Classes 2 and 3 could be obtained from the capacitance measurement of Classes 3 and 4, respectively. The ESR according to Class 4 would have required a current which was not feasible, so it was omitted. The leakage current was measured as the current into the device when it had been held at 0.9 V for 30 min; thus it could be recorded from the galvanostatic measurements, before the beginning of the discharge.

### Solar cell fabrication and characterization

The OPV module was prepared on indium tin oxide-poly(ethylene terephthalate (ITO-PET) substrate. The patterning of ITO and the deposition of a hole transporting layer (poly(3,4-ethylenedioxythiophene): poly(styrenesulfonate) known as PEDOT:PSS) and a photoactive layer (poly-3-hexylthiophene: [6.6]-phenyl-C61-butyric acid methyl ester known as P3HT:PCBM) were fabricated using roll-to-roll printing processes. On top of the photoactive layer, lithium fluorine and aluminium were thermally evaporated through a shadow mask and finally encapsulated with barrier-adhesive foil. One module with a size of 15 cm^2^ comprised eight serially connected cells. The OPV module fabrication has been reported in detail by Apilo *et al.*^35^. The performance of OPV modules (with encapsulation) under AM1.5 illumination of 1 sun was as follows: open circuit voltage of 4.5–4.6 V (8 cells in series), short-circuit current of 16 mA and a power conversion efficiency of 1.6–1.8%. The current-voltage (I–V) curve for two OPV modules used in this work are shown in Supplementary Information Fig. S1. The OPV module 315 was used in the SC self-discharge experiment and the module 385 in the ECD operation demonstrator.

### Measurement setup

The voltage of the SC was monitored in all experiments using an USB-multimeter (NI USB-4065 USB DMM, Low-Cost 6.5 Digit USB Digital Multimeter). The multimeter has an input resistance of 1 GΩ which has to be taken into account in the SC self-discharge measurements. Voltage measurement data was recorded using a LabVIEW program. The electrochromic display element used in the display operation experiment was obtained from Ynvisible.

In the energy harvesting experiments, the OPV modules were exposed to two different fluorescent lighting sources; ceiling lighting (fluorescent tubes) or a desk lamp (Osram Dulux S 11 W/827^37^). The desk lamp was placed above the solar cell on the distances of 20 and 40 cm while in the ceiling lighting experiment the OPV module was placed on the office desk. The illuminance level in each lighting condition was measured using a lux-meter FieldMaxII-TOP Laser Power/Energy Meter from Coherent Inc. (see Supplementary information Fig. S3).

### Electron microscopy

The electron microscopy analysis of the CNT/xylan nanocomposite ink was performed using a field-emission scanning electron microscope (FESEM, Zeiss ULTRAplus) and transmission electron microscope (TEM, Jeol JEM-2010) equipped with energy dispersive X-ray spectrometer (EDS, Noran Vantage with Si(Li) detector, Thermo Scientific). For FESEM imaging, a drop of the ink was places on the aluminum pin stub, whereas for SEM imaging, a drop of the ink was placed on the copper grid with a holey carbon support film. The ink drops were then let to dry in air at room temperature.

## Additional Information

**How to cite this article**: Tuukkanen, S. *et al.* Behaviour of one-step spray-coated carbon nanotube supercapacitor in ambient light harvester circuit with printed organic solar cell and electrochromic display. *Sci. Rep.* **6**, 22967; doi: 10.1038/srep22967 (2016).

## Supplementary Material

Supplementary Information

## Figures and Tables

**Table 1:** Summary of the CNT supercapacitor performance from the galvanostatic discharge measurements according to an industrial standard IEC 62391-1 30.

Class	Application	*I*_*meas*,*C*_ (µA/cm^2^)	*C* (mF)	*C*_*areal*_ (mF/cm^2^)	*C*_*sp*_ (F/g)	*I*_*meas*,*ESR*_ (µA)	ESR (Ω)
2	Energy storage	1.1	81	2.7	1.5	340	46
3	Power	11.2	93	3.1	1.7	3400	37
4	Instantaneous power	112.4	89	3.0	1.6	–	–

The table shows the measurement classification and corresponding application, device capacitance (*C*), area specific capacitance (*C*_*areal*_), mass specific capacitance (*C*_*sp*_), equivalent series resistance ESR, as well as the used discharge currents for *C* and ESR measurements specified in the standard (*I*_*meas*,*ESR*_ = 10 × *I*_*meas*,*C*_).

**Table 2:** Charging of the SC by the OPV module under ambient lighting and subsequent SC self-discharge.

Light source	Illuminance (lx)	*T*_*light*_ (K)	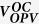 (V)	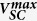 (V)	*t*_*ch*_ (min)	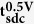 (min)	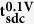 (h)
Ceiling lighting	388	2924	1.05	0.92	171	56.0	3.57
Desk lamp at 40 cm	1585	2761	2.02	1.02	20.2	57.9	5.05
Desk lamp at 20 cm	5985	2713	3.10	1.00	4.18	35.4	5.54

In addition to the light illuminance and temperature levels for different ambient lighting conditions, the open circuit voltage (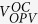) of the OPV module under the given lighting conditions, the voltage level to which the SC was charged (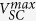), the charging times (*t*_*ch*_) and the self-discharging times to 0.5 V (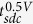) and 0.1 V 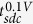 are presented.

**Table 3:** The electrochromic display operation using the supercapacitor charged with OPV under ambient lighting.

Working environment	illuminance (lx)	*T*_*light*_ (K)	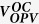 (V)	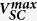 (V)	*t*_*ch*_ (min)	Δ*V*_*sc*_ per ECD update (mV)
Filing, copying, etc.	300	3001	1.62	0.75	61.6	15
writing, typing, reading, data processing	500	2885	2.06	0.85	48.8	20
Technical writing	750	2849	2.41	0.85	41.8	36

The light illuminance and temperature levels for different lighting conditions, open circuit voltage (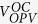), maximum voltage to which the supercapacitor was charged (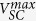), charging time (*t*_*ch*_) and supercapacitor voltage drop per one display state change operation Δ*V*_*sc*_.
