# Supplementary material for: Behaviour of one-step spray-coated carbon nanotube supercapacitor in ambient light harvester circuit with printed organic solar cell and electrochromic display
Source: Sci Rep. 2016 Mar 9;6:22967. doi: 10.1038/srep22967 (PMC4783710; doi:10.1038/srep22967)
Supplement: Supplementary Information [file srep22967-s1.pdf]

Supplementary Information

Behaviour of one-step spray-coated carbon nanotube supercapacitor in ambient light harvester circuit with printed organic solar cell and electrochromic display

Sampo Tuukkanen[1,*], Marja Välimäki[2], Suvi Lehtimäki[3], Tiina Vuorinen[3] and Donald Lupo[3]

[1] Tampere University of Technology, Department of Automation Science and Engineering, P.O. Box 692, FI-33101 Tampere, Finland

[2] VTT Technical Research Centre of Finland, P. O. Box 1100, FI-90570 Oulu, Finland

[3] Tampere University of Technology, Department of Electronics and Communications Engineering, P.O. Box 692, FI-33101 Tampere, Finland

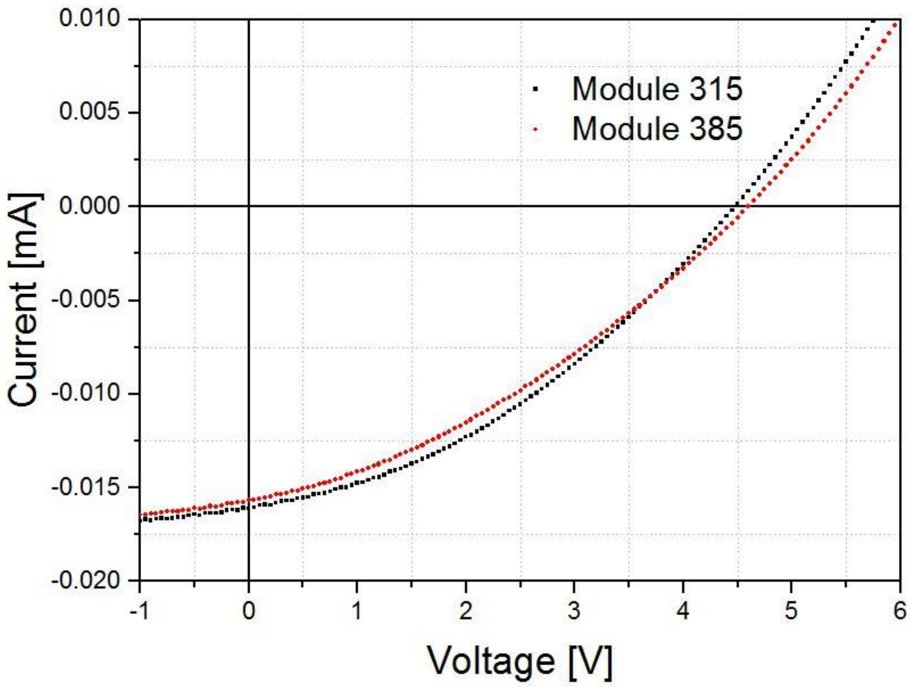

Figure S1. The current-voltage characteristics of two OPV modules used in this work. OPV module fabrication and characterization details are reported in [1].

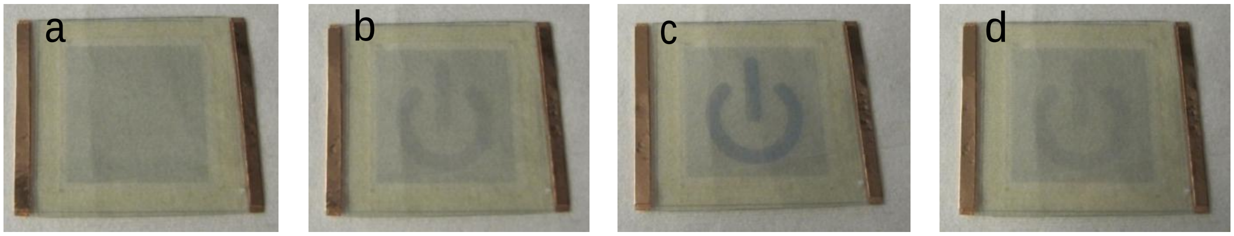

Figure S2. Electrochromic display (ECD) switching behavior and stability. External voltage supply was for switching here. a) Display before the experiment (in OFF state). b) Display switched ON using 0.5 V potential. c) Display switched ON using 1 V potential. d) Display after 5 minutes waiting from switching at 1V. The ECD was obtained from Ynvisible.

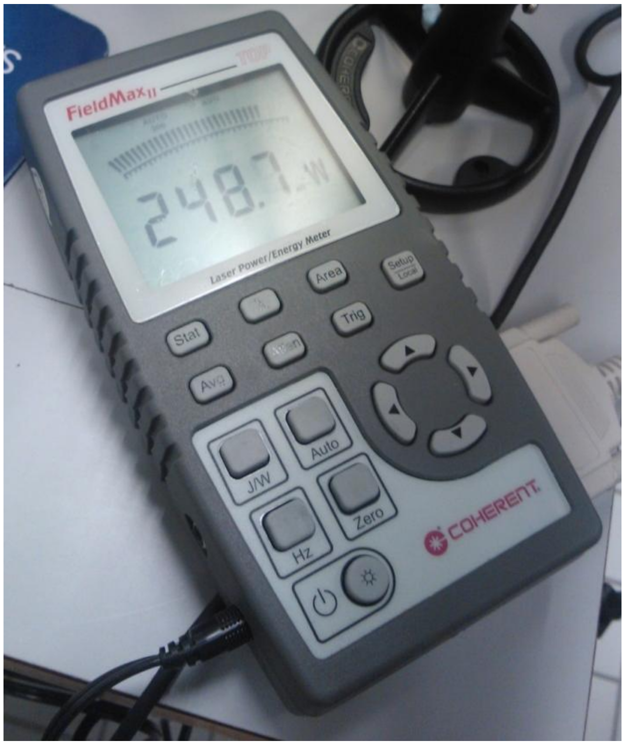

Figure S3. Photograph of the LUX-meter used for illuminance measurement in this work.

Supplementary information references

[1] Apilo, P., Hiltunen, J., Välimäki, M., Heinilehto, S., Sliz, R., & Hast, J. (2014). Roll-to-roll gravure printing of organic photovoltaic modules—insulation of processing defects by an interfacial layer. Progress in Photovoltaics: Research and Applications.
